# Resin infiltration versus fluoride varnish for visual improvement of white spot lesions during multibracket treatment. A randomized-controlled clinical trial

**DOI:** 10.1007/s00784-024-05695-2

**Published:** 2024-05-11

**Authors:** Yamen Kashash, Sascha Hein, Gerd Göstemeyer, Pervin Aslanalp, Manon Isabelle Weyland, Theodosia Bartzela

**Affiliations:** 1 Depatment of Orthodontics and Dentofacial Orthopedics, Charité – Universitätsmedizin Berlin, Aßmannshauser Straße 4-6, 14197 Berlin, Germany; 2 School of Design, University of Leeds, Woodhouse Lane, Leeds, LS2 9JT England; 3 Department of Conservative, Preventive and Pediatric Dentistry, Charité – Universitätsmedizin Berlin, Aßmannshauser Straße 4-6, 14197 Berlin, Germany; 4 Department of Orthodontics, University Hospital Carl Gustav Carus Dresden, Technische Universität Dresden, Fetscherstraße 74, 01307 Dresden, Germany

**Keywords:** White spot lesions, Demineralization, Resin infiltration, Orthodontics, Fluoride, Multibracket appliance

## Abstract

**Aims:**

This study aimed to evaluate the visual improvement of resin infiltration of white spot lesions (WSL) during orthodontic treatment with the multibracket appliance (MBA) compared to fluoride varnish.

**Methods:**

Patients aged 12–17 years with at least one WSL with an International Caries Detection and Assessment System (ICDAS) score of 1–2 during an active MBA treatment were included and randomized to receive either resin infiltration (Icon) or fluoride application (Flairesse). Standardized digital images were obtained before, one-day, one-week, one-month, three-months and six-months after treatment using a DSLR camera and a matching polarization filter. A grey reference card was used for color standardization. A Matlab routine was used to measure the color difference between adjacent healthy enamel and treated WSL. The independent-samples t-test was used for intergroup and paired-samples t-test for intragroup comparison.

**Results:**

Images of 116 teeth from 36 patients were analyzed. The ΔE for the “Icon” treated WSL was smaller (T1_ICON_ = 5.0 ± 1.4) than in the fluoride group (T1_Fluoride_ = 8.4 ± 3.2). Caries infiltration significantly improved the aesthetic appearance of WSL (*p* < 0.001), which remained satisfactory at six months (T5_ICON_ = 5.2 ± 1.6).

**Conclusion:**

WSL infiltration management during orthodontic treatment was superior to topical fluoridation in not only arresting the enamel lesions but also significantly improving the aesthetic appearance of demineralized regions around the brackets.

**Clinical relevance:**

WSL treatment in orthodontic patients is usually initiated after debonding. Research has shown that the earlier WSL is treated, the better the aesthetic outcome. There is limited data on the efficacy of resin infiltration of WSL during orthodontic treatment.

**Supplementary Information:**

The online version contains supplementary material available at 10.1007/s00784-024-05695-2.

## Introduction

Multibracket appliance (MBA) has been established as a standard orthodontic treatment. One side effect of this treatment is that iatrogenic plaque retention sites are created, leading to increased accumulation of pathogenic biofilms in the area adjacent to the appliances [1]. In addition to plaque-induced gingivitis [2], this can lead to carious white spot lesions (WSL). The prevention of WSL during orthodontic treatment with MBA is of paramount importance to orthodontists [3], as up to 40% of orthodontic patients, or even twice as many (79.3%) as recently reported in an academic setting, may develop WSL during the first six months of treatment [4, 5]. This is acknowledged as a significant concern in orthodontics and requires preventive measures to reduce the risk.

WSL exhibit a decrease in mineral density and mechanical properties of the affected enamel, along with changes in molecular composition and surface microstructure [6]. Compared to healthy enamel, the whiter appearance results from strong light scattering within the lesion. This is essentially because the mineral particles in the lesion are surrounded by water instead of mineral-rich enamel. The higher water content in lesions creates significant differences, resulting in shorter photon paths, reduced absorption, and lower transparency. This increased light absorption is mainly due to the increased variation in refractive index between the lesion substance and its surroundings [7].

Fluoride-containing products in their various administration forms are widely used to prevent and treat WSL [8, 9] with varying success rates [10]. In addition to fluoride treatment, resin infiltration is an effective and valid method for treating WSL. This approach not only enhances the tooth’s aesthetics but also helps arrest the early stages of tooth decay [11, 12].

One of the advantages of resin infiltration is that, due to the similar refractive index of the infiltrated and healthy enamel areas, the resin appears as a hard tooth substance, blending seamlessly with the natural tooth structure. Clinical evidence suggests that resin infiltration of WSL results in improved appearance and preservation of tooth structure, making it a desirable treatment option for WSL [11–16]. The mean color difference between WSL and sound adjacent enamel (SAE) at six months was significantly smaller in the resin infiltration group than in the fluoride group (5.64 vs. 7.59 Δ*E*) [13]. Moreover, the color assimilation of the infiltrated WSL to the SAE remained stable even 24 months after treatment [14]. Evidence further indicates that the best aesthetic outcome is achieved by treating the WSL as soon as possible after brackets debonding [13]. One approach to further improve the aesthetic outcome of the resin infiltration technique might be to treat the WSL prior to bracket removal. However, the feasibility of this approach has not been adequately investigated and warrants further research to determine its efficacy and practicality.

The present randomized-controlled clinical trial aimed to investigate the efficacy and durability of the aesthetic outcomes, measured by changes in color and lightness of WSL, after resin infiltration compared to fluoride varnish application during fixed orthodontic treatment. The study tested the null hypothesis of no significant color difference (WSL vs. SAE) between the resin infiltration and fluoride varnish groups six months after intervention.

## Material and methods

This trial was registered at the German Clinical Trials Register (DRKS-ID: DRKS00027344). Reporting of this trial follows the CONSORT 2010 statement [17]. The study protocol was approved by the Ethics Committee at the Charité-University Berlin (EA2/289/20), and informed consent was obtained from all patients or legal guardians before enrollment. This is a multicenter, two-arm, randomized-controlled trial with a parallel group design.

Patients younger than 17 years of age undergoing orthodontic treatment with metal MBA were eligible for inclusion. All patients had to have fully erupted and unrestored permanent maxillary and mandibular canines and incisors and at least one WSL graded 1 to 2 according to the International Caries Detection and Assessment System (ICDAS) [18] on the labial surface of the previously mentioned teeth. Exclusion criteria included cavitated lesions, filled or restored teeth, enamel abnormalities, and deciduous teeth. The primary endpoint was set at six months from baseline.

Sample sizes were determined using estimates from prior studies assessing the effectiveness of resin infiltration of WSL, relying on color differences computed from the CIELAB color space [13]. Assuming a standard deviation of 3, an effect size of 3.61, and a two-tailed test with an alpha error of 0.05, 16 patients were required in each group to provide 90% statistical power. To account for potential patient dropouts and non-compliance with the study protocol, a total of 38 patients were recruited into the trial.

Prior to the trial, opaque envelopes were prepared containing unique participant allocation assignments based on a computer-generated random sequence. This allocation sequence was generated before the trial by an independent assistant. The assignment details were unknown to the operators or the coordinator. The sealed envelopes were securely stored in a location inaccessible to the researchers. On the day of treatment, the patient drew an envelope in the presence of a dental assistant. The sealed envelope was then opened to reveal the intervention allocation (resin infiltration or fluoride varnish application). Afterward, the dental assistant prepared the instruments for the allocated treatment, and the dentist was invited into the treatment room.

Blinding of operators was not possible due to the nature of the treatment. Study participants were blinded until the allocation. Nonetheless, the outcome assessors and the statistician were kept blinded, as they did not have access to the randomization list and were presented with images for evaluation marked solely with subject numbers.

First, orthodontic wires and auxiliaries were removed for better access to the working field, and the affected teeth were cleaned with a fluoride-free prophylaxis paste (Cleanic, KerrHawe, Bioggio, Switzerland). The teeth were isolated with a light-curing liquid rubber dam (Opaldam, Ultradent, Cologne, Germany) to protect the soft tissues from the chemicals used and create a dry working field. Caries infiltration was carried out strictly according to the manufacturer's instructions, with up to three etching procedures (Icon Infiltrant, DMG, Hamburg, Germany). The surface of the lesion was etched by applying a 15% HCl gel (Icon-Etch, DMG, Hamburg, Germany) for 120 s. Subsequently, the HCl gel was rinsed for 30 s and dried with oil-free air. The lesion was then dried with ethanol (Icon-Dry, DMG, Hamburg, Germany) to remove water within the micropores and to ensure adequate penetration of the infiltrant into the depth of the lesion. The infiltrant was then applied to the WSL for 180 s. Excess material was removed, and the infiltrant was light cured for 60 s using an LED light held as close as possible to the restoration. The infiltrant was applied a second time for 60 s, and the excess was removed before curing for a final time for 60 s. Finally, polishing brushes were applied with light pressure (Occlubrush, Kerr, Orange, USA). A 5% fluoride varnish (Flairesse, DMG, Hamburg, Germany) was used in the control group. Following the removal of the orthodontic wires and auxiliaries, the teeth were isolated with cotton rolls. A thin layer of fluoride varnish was applied with the disposable brush applicator, and the patients were instructed not to eat or drink for one hour. This varnish was continuously used in the control group twice a month.

The final color stability of the treated WSL was assessed through reflected, cross-polarized dental photographic documentation [19]. Tooth color was recorded before exposure to air, as dehydration of teeth has been shown to result in a statistically significant increase in whiteness after two minutes [20].

The images were captured using a Nikon D3200 digital camera equipped with a macro lens (AF-S DX Micro Nikkor 85mm f/3.5G ED VR, Nikon Corp., Japan), and a ring flash (Meike 14EXT i-TTL, Meike Global, China) with a matching polarizing filter (polar_eyes, Emulation, Freiburg, Germany) attached to it. All photographs were taken under consistent ambient light conditions using the same camera setup, adhering to the eLAB protocol [21]. This protocol specifies an exposure time of 1/125 s, an aperture of f/22, an ISO setting of 100, and RAW image quality. All photos were captured by the same investigator.

Subsequently, the images underwent automatic calibration in the eLAB_prime software (Emulation, Freiburg, Germany) and were then exported as PNG files to Photoshop CS6 (Adobe, San Jose, USA) for cropping and superimposition. To evaluate color stability, the template matching technique [22] was employed in MATLAB (R2022a, MathWorks, Germany).

**Fig. 1:**
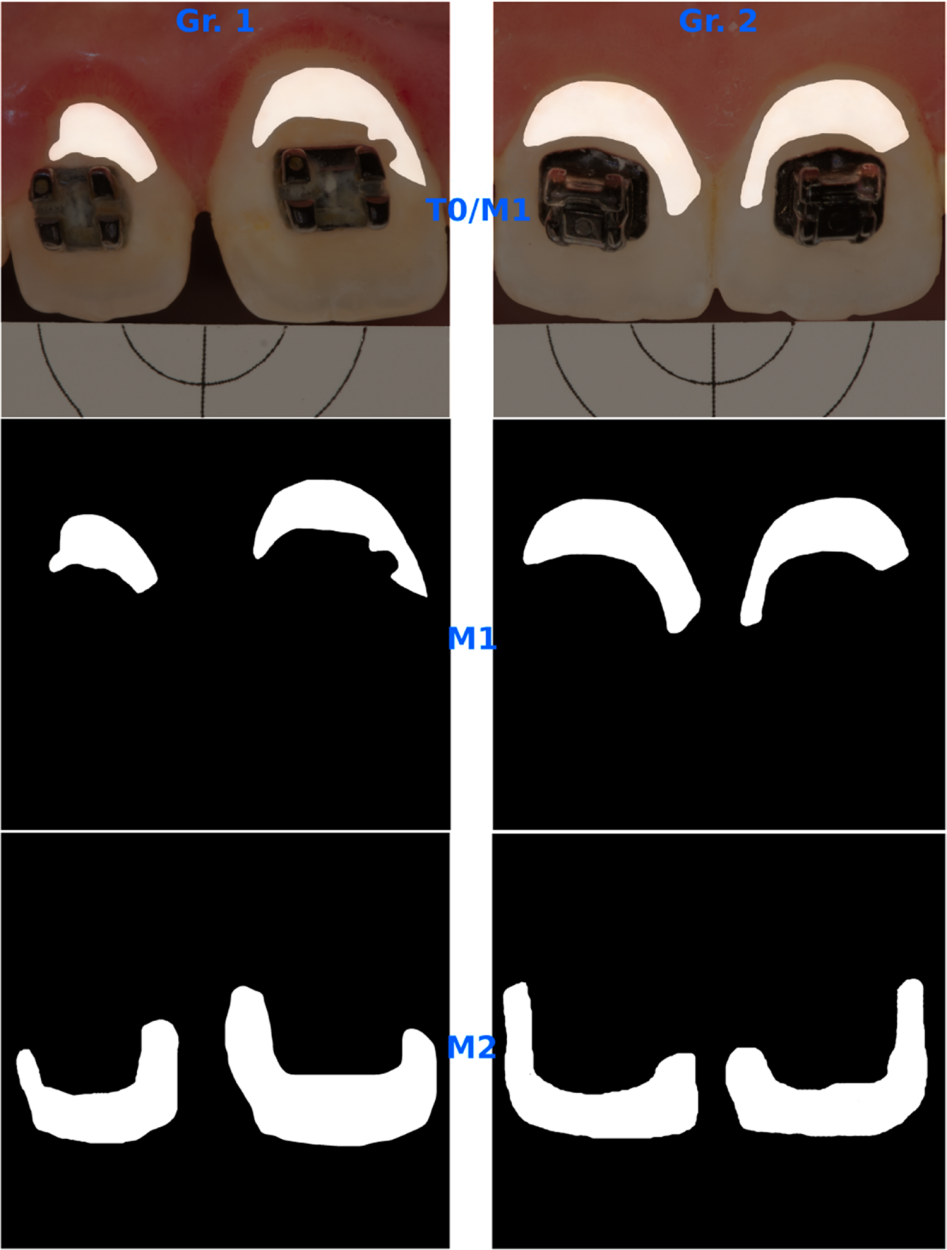
Images of the two black and white masks; M1 and M2, representing affected and sound enamel, respectively. Gr. 1: Icon-group, Gr. 2: Fluoride-group, T0: baseline

**Fig. 2:**
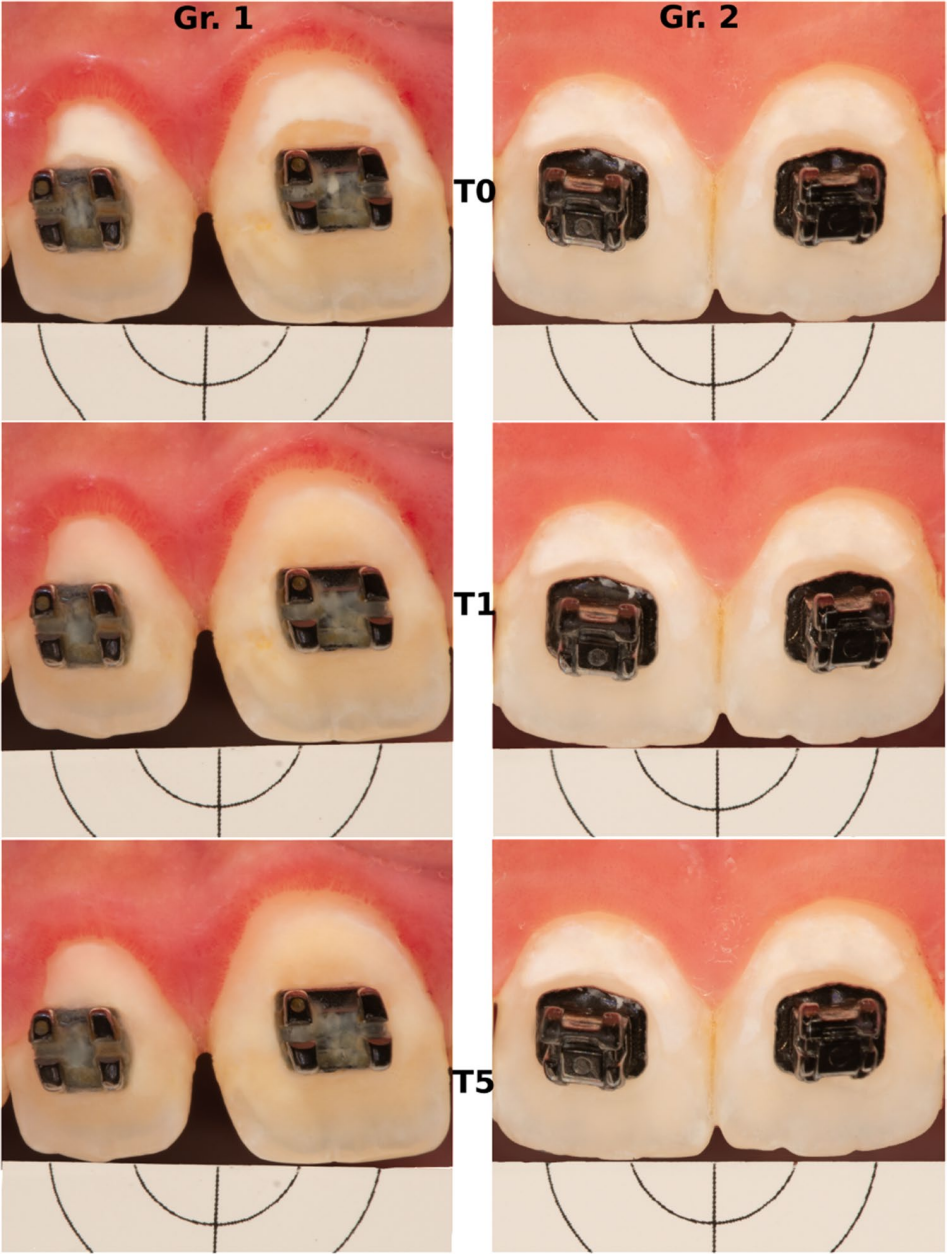
Images of two subjects. Gr. 1: Icon-group, Gr. 2: Fluoride-group, T0: baseline, T1: 1st day of observation, T5: 6 months later

Initially, two black and white masks, referred to as M1 and M2 (Fig. 1), were generated to isolate regions of interest representing WSL and SAE, respectively. The CIEDE2000 color difference equation (∆*E*_00_) [23] was utilized to systematically compare these templates with intraoral images taken at different stages, including the day of treatment (T0), one day (T1), one week (T2), one month (T3), three months (T4) and six months later (T5), as depicted in Fig. 2. In addition, the ICDAS score was recorded at T0 and T5.

Oral hygiene was evaluated based on the sulcus bleeding index (SBI) [24] and the accumulation of plaque using the visible plaque index (VPI) [25]. Furthermore, the periodontal assessment of participants’ conditions were made using the periodontal Screening Index (PSI) [26]. Assessment was performed before preparing the teeth for treatment (T0) and six months later (T5).

### Statistical analysis

Statistical analysis of the data obtained was performed using IBM SPSS Statistics version 28 (SPSS Inc., Chicago, IL, USA). Descriptive statistics, including mean values, standard deviations, and minimum and maximum values, were calculated for both groups. The independent-samples t-test was employed to compare between the two groups, while the paired-samples t-test was utilized to compare within the groups. The significance level was set at α = 0.05 for all statistical tests.

## Results

Eligible participants were recruited from September 2022 to July 2023 at the Department of Orthodontics and Dentofacial Orthopedics, Charité-Universitätsmedizin Berlin, and one private practice in Berlin. Interventions and follow-up visits took place from October 2022 to December 2023. Recruitment was stopped in July 2023 as the number of allocated participants (*N* = 38) reached the calculated sample size.

**Fig. 3:**
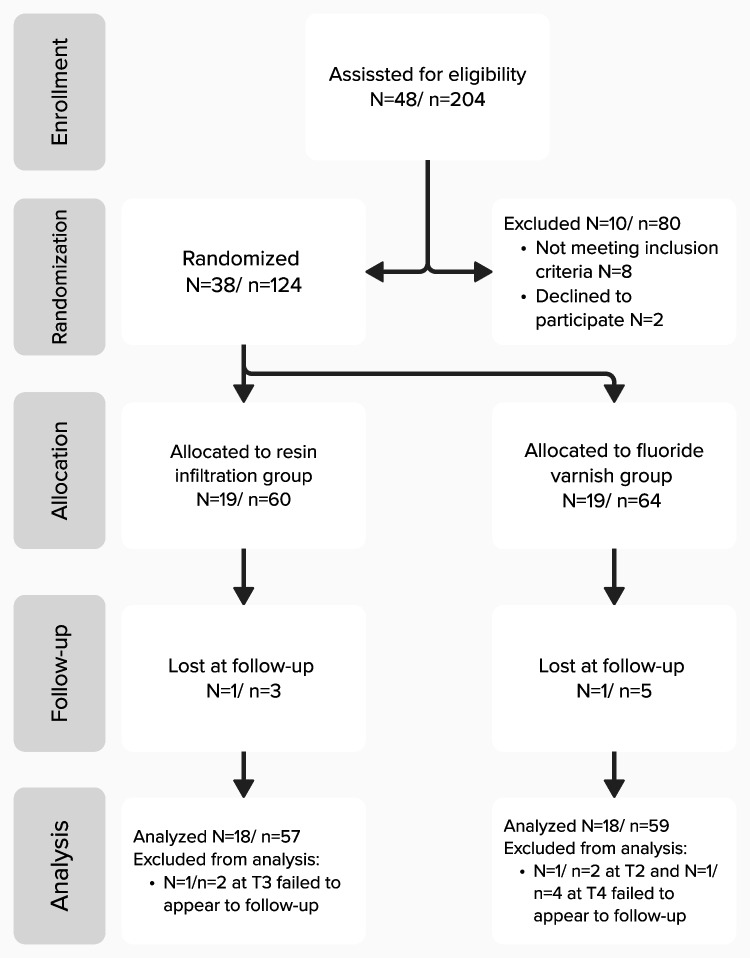
Flowchart of the participant. *N* number of subjects, *n* number of teeth, *T1* 1st day of observation, *T2* 1 week later, *T3* 1 month later, *T4* 3 months later

Figure 3 shows a flowchart of the participant. Thirty-eight participants were recruited. Two participants were excluded from the study after failing to attend all follow-up appointments. Thirty-six subjects aged 12–17 years (116 teeth) were equally distributed to each group (fluoride-group: 59 teeth, Icon-group: 57 teeth).

**Fig. 4:**
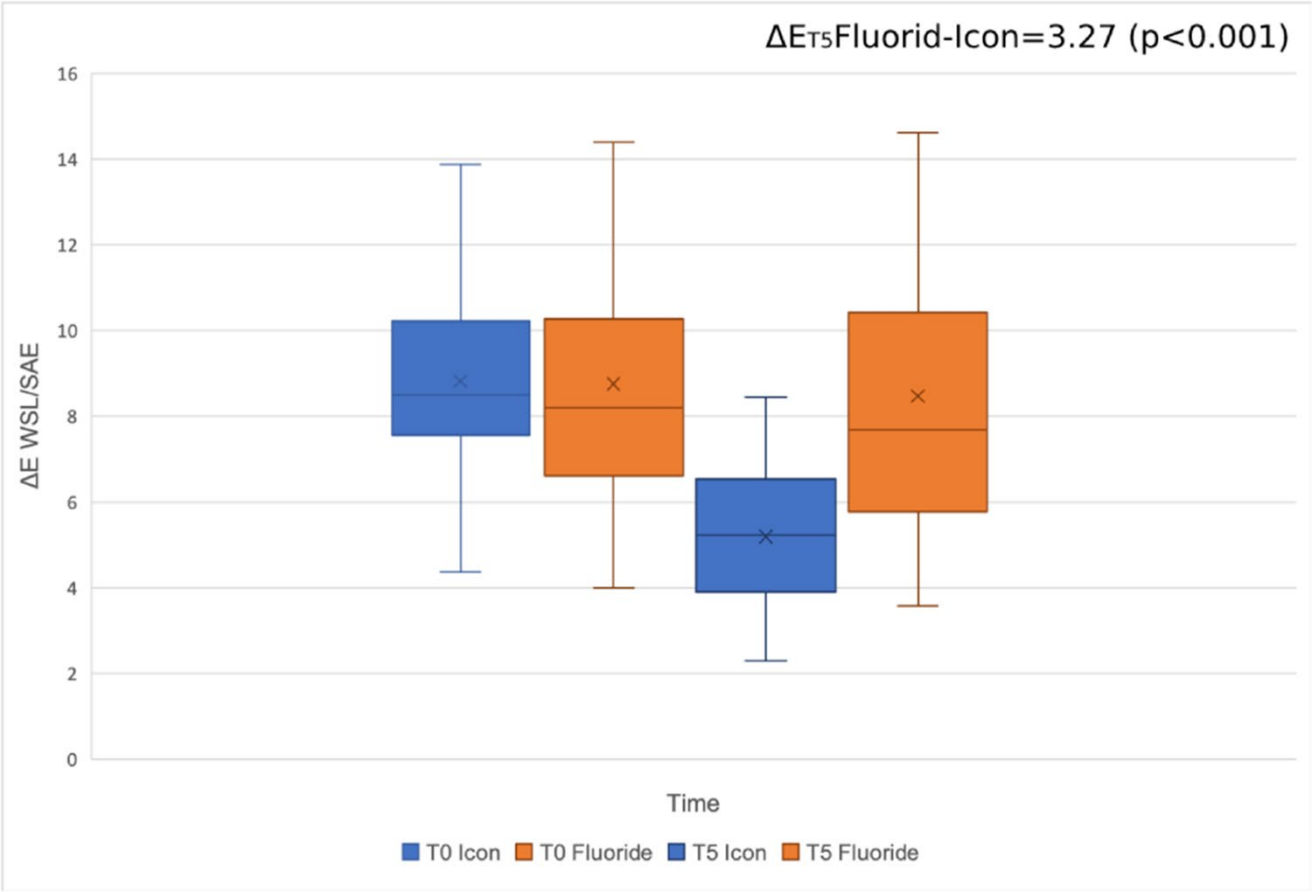
Summarized ∆*E* of white spot lesion (WSL) and sound adjacent enamel (SAE) before treatment (T0) and after six months (T5). In contrast to the resin infiltration group, no significant changes were observed in the fluoride varnish group

At baseline, there were no significant differences in the demographic and clinical characteristics between the two groups, as indicated by the values presented in Table 1 (Icon ΔE_T0_: 8.81 ± 2.17 vs. Fluoride ΔE_T0_: 8.74 ± 3.21). At T1, the color difference between SAE and WSL showed a significant reduction in the resin infiltration group ΔE_T1_: 5.01 ± 1.45. These changes remained stable after six months, ΔE_T5_: 5.19 ± 1.60 (Table 2). In the fluoride varnish group, there were no significant differences in ΔE values between baseline and one day after treatment, ΔE_T0-T1_: 0.25 ± 0.53, which remained stable throughout the observation period of one week, one month, three months, and six months after treatment (Table 2). At T5, a statistically significant color difference of 3.27 CIELAB units (*P* < 0.001) was observed between the infiltration group ΔE_T5_: 5.19 ± 1.60 and the fluoride group ΔE_T5_: 8.46 ± 3.52 (Fig. 4).

**Table 1:** Demographic and clinical characteristics between the two groups at baseline

		Icon (*n* = 57)	Fluoride (*n* = 59)
Age mean		14.4	14.1
Gender	Female	8	7
	Male	10	11
Localization	Upper	42	47
	Lower	15	12
Δ*E* mean ± SD		8.81 ± 2.17	8.74 ± 3.21
ICDAS mean		1,89	1,83

*n* number of teeth, *ΔE* color difference, *ICDAS* International Caries Detection and Assessment System.

**Table 2:** Development of Δ*E*_00_ values between white spot lesion and sound adjacent enamel in both groups

Group	Time	*n*	Δ*E*_00_ mean	SD	Time	Δ*E*_00_ mean	SD	*P* value
Icon	T0	59	8.81	2.17				
	T1	59	5.01	1.45	T0-T1	3.80	2.11	< 0.001*
	T2	57	5.16	1.55	T0-T2	3.65	2.22	< 0.001*
	T3	59	5.40	1.57	T0-T3	3.56	2.01	< 0.001*
	T4	55	5.23	1.51	T0-T4	3.57	2.10	< 0.001*
	T5	59	5.19	1.60	T0-T5	3.62	2.00	< 0.001*
Fluoride	T0	57	8.74	3.21				
	T1	57	8.48	3.16	T0-T1	0.25	0.53	< 0.001*
	T2	57	8.72	3.21	T0-T2	0.10	0.57	0.165
	T3	55	8.66	3.32	T0-T3	0.08	0.78	0.425
	T4	57	8.41	3.42	T0-T4	0.21	0.81	0.059
	T5	57	8.46	3.52	T0-T5	0.28	0.82	0.10

*T0* Baseline, *T1* 1 day, *T2* 1 week, *T3* 1 month, *T4* 3 months, *T5* 6 months, *n* number of teeth, *ΔE*_*00*_ color difference. *P < 0.05.

No clinically relevant changes were observed in the SAE in terms of changes in lightness (∆L*) and changes in the red-green (∆a*) or blue-yellow (∆b*) color axes (Table 3).

**Table 3:** Intergroup comparisons of L*, a*, b*, and ∆*E*_00_ values at baseline, one day and six months after treatment in both groups

Time	Parameter	Area	Icon: Mean ± SD	Fluoride: Mean ± SD	Mean diff	95% CI	*P* value
T0	L*	WSL	76.80 ± 7.19	76.38 ± 8.34	0.42	-2.44–3.28	0.77
	L*	SAE	67.02 ± 6.01	68.37 ± 6.28	-1.35	-3.63–0.90	0.23
	a*	WSL	5.25 ± 4.16	6.05 ± 3.19	-0.8	-2.16–0.57	0.25
	a*	SAE	6.18 ± 3.65	5.98 ± 2.88	0.2	-1.01–1.41	0.74
	b*	WSL	10.97 ± 6.85	10.91 ± 4.76	0.06	-2.12–2.24	0.95
	b*	SAE	14.45 ± 7.15	14.79 ± 6.78	-0.45	-2.90–2.22	0.79
	Δ*E*	WSL/SAE	8.81 ± 2.17	8.74 ± 3.21	0.07	-0.94–1.07	0.89
T1	L*	WSL	68.36 ± 5.95	75.80 ± 8.79	-7.44	-9.19 to -3.67	< 0.001*
	L*	SAE	67.06 ± 6.06	68.58 ± 5.99	-1.52	-3.74–0.69	0.17
	a*	WSL	8.82 ± 4.86	6.62 ± 3.35	2.2	1.06–4.13	0.001*
	a*	SAE	6.12 ± 3.43	5.89 ± 2.84	0.23	-0.93–1.39	0.69
	b*	WSL	16.15 ± 7.58	11.33 ± 4.94	4.82	2.44–7.18	< 0.001*
	b*	SAE	14.38 ± 6.85	14.82 ± 6.75	-0.44	-2.93–2.06	0.73
	Δ*E*	WSL/SAE	5.01 ± 1.45	8.48 ± 3.16	-3.47	-4.38 to -2.57	< 0.001*
	Δ*E* T0 vs. T1	WSL/SAE	3.80 ± 2.11	0.25 ± 0.53	3.55	2.97–4.13	< 0.001*
T5	L*	WSL	69.59 ± 5.70	75.45 ± 8.77	-5.86	-8.58 to -3.14	< 0.001*
	L*	SAE	66.26 ± 10.02	68.45 ± 6.18	-2.19	-5.30–0.93	0.16
	a*	WSL	8.74 ± 4.94	6.43 ± 3.42	2.31	0.73–3.88	0.004*
	a*	SAE	5.86 ± 3.37	5.87 ± 2.96	-0.01	-1.18–1.15	0.97
	b*	WSL	15.83 ± 7.64	11.60 ± 4.82	4.23	1.86–6.59	< 0.001*
	b*	SAE	14.02 ± 6.84	14.77 ± 6.88	-0.75	-3.27–1.77	0.55
	Δ*E*	WSL/SAE	5.19 ± 1.60	8.46 ± 3.52	-3.27	-4.27 to -2.26	< 0.001*
	Δ*E* T0 vs. T5	WSL/SAE	3.62 ± 2.00	0.28 ± 0.82	3.34	2.76–3.90	< 0.001*

*T0* Baseline, *T1* 1 day, *T5* 6 months, *WSL* white spot lesion, *SAE* sound adjacent enamel, L* Lightness value, a* color coordinate on the green–red axis, b* color coordinate on the blue-yellow axis, *ΔE*_*00*_ color difference. *P < 0.05.

At T5, the mean ICDAS score in the resin group was 0.40, significantly lower than the fluoride group score of 1.61 (*p* < 0.001). There were no significant differences in oral hygiene between the two groups at baseline and six months, based on the indices assessed (PSI, SBI, VPI).

## Discussion

This clinical trial evaluated the short-term in vivo color stability of resin infiltration treatment in patients receiving orthodontic treatment with MBA compared to treatment with fluoride varnish. Color stability was assessed six months after treatment of WSL on 116 permanent teeth in 36 patients. Following the treatment, the resin infiltration group exhibited a significant reduction in the color differences between WSL and SAE compared to the fluoride group. The color difference remained consistent at the six-month follow-up in the infiltration group, indicating excellent short-term color stability. The null hypothesis of no significant color difference Δ*E*_WSL-SAE_ between the infiltration and fluoride groups after six months was rejected. Our results are consistent with previous studies [12, 13, 15, 16].

Even though the SAE was also infiltrated, there were no clinically relevant color changes. This finding is in agreement with previous studies [13, 27]. The use of resin infiltration to arrest and mask WSL has been reported in several in vitro and in vivo studies. However, this type of microinvasive WSL treatment during orthodontic treatment, although recently widely reported [28], is still not employed in everyday dental practice. In this study, patients were admitted immediately after initial diagnosis, which may be advantageous in preventing further progression of the lesions by waiting until after debonding to initiate treatment, as was done in previous studies [14, 15, 29].

Inadequate oral hygiene leading to biofilm accumulation, especially around brackets, a diet rich in refined carbohydrates, and frequent carbohydrate intake are the main factors in the development of WSL [30, 31]. This highlights the role of oral hygiene instruction, nutritional counselling and regular dental check-ups during orthodontic treatment in preventing WSL and minimizing their incidence. Although some spontaneous improvement can be expected after the removal of MBA [32], WSL still pose an aesthetic concern. A widespread practice is the treatment of WSL that occur during orthodontic treatment by local fluoridation [33]; this approach fails to address the aesthetic problem and subjects the decalcified areas to abrasion during brushing, leading to further loss of tooth structure [34].

One approach to further improve the aesthetic outcome of the resin infiltration and stop the progression of WSL during orthodontic treatment with MBA could be infiltrant implementation prior to bracket removal. Evidence suggests that the earlier the treatment is initiated, the better the aesthetic outcomes [13, 35]. The present findings demonstrate that resin infiltration can be effectively used as a microinvasive treatment of WSL during orthodontic treatment with MBA. Furthermore, the early resin infiltration intervention would prevent the premature forced interruption of the orthodontic treatment, leading to compromised orthodontic outcomes. Long-term follow-up is required to confirm the results, as well as post-debonding evaluation to assess the overall aesthetic improvement and the implementability of such an approach in a protocol for the prevention and treatment of WSL.

Quantification techniques for analyzing WSL in dentistry encompass a variety of instruments, with colorimeters, spectrophotometers, and digital imaging systems being the most prominent [36]. Due to its convenience, the contact photo spectrometer, exemplified by devices like the Vita Easy Shade, has gained popularity in dental research. However, it is limited to single-spot measurements within a fixed observation window, rendering it unsuitable for the discrete assessment of WSL, which often appear as irregularly shaped patches requiring differentiation from their surroundings.

Prior research related to WSL has primarily employed either digital imaging systems [12, 28, 37] or multispectral cameras [13, 15, 16]. Each approach offers distinct advantages and disadvantages. Calibrated camera systems like eLAB aim to capture consistent red, green, and blue (RGB) values over time and establish a reasonably linear relationship with the International Commission on Illumination (CIE) XYZ tristimulus values with acceptable accuracy [38]. They enable non-contact tooth color measurement at a relatively low cost and can be combined with editing tools and additional software. However, they necessitate strict adherence to a photographic protocol, demanding rigorous discipline that may not always align with the realities of research settings. On the other hand, multispectral cameras blend the advantages of imaging systems with pixel-wise spectral reflectance measurements [39]. Regrettably, the two devices most frequently mentioned in WSL-related research have been largely discontinued, leaving a void in this particular measurement modality. As a result, researchers face challenges in finding suitable instruments for the comprehensive assessment of WSL in contemporary studies.

We correlated color changes of the entire WSL using standardized digital images, whereas the other study, which reported WSL treatment during orthodontic treatment, correlated color changes at the most prominent sites within the enamel lesion [28]. Therefore, a direct comparison of the results is not possible. In addition, laser fluorescence readings were taken with DIAGNOdent. This approach was not used in this study because it has been reported that dental materials such as infiltrants can interfere with DIAGNOdent readings, leading to incorrect interpretations. In addition, it was not always possible to measure lesions detected by visual inspection [40, 41].

A limitation of this study is that the area of SAE used as a reference was incisal, resulting in a primary color difference between the areas compared (WSL/SAE) [42]. The selection of a more adjacent area, such as the intact tooth surface under the brackets, was not possible due to the nature of the study. Although a split-mouth design is often preferred in studies of caries infiltrates following orthodontic treatment, the parallel group design was chosen for this study to avoid bias on aesthetic outcomes. In addition, for ethical reasons, it was not possible to leave the control group untreated, so both groups had to receive treatment in this trial. Furthermore, T1 was not performed on the day of treatment, but the following day to avoid color changes caused by tooth isolation and subsequent dehydration [43, 44]. A further limitation is the lack of data after debonding to evaluate the treatment outcome in the area around the bracket. Follow-up of the participants is planned after the removal of the MBA.

## Conclusions

The findings suggest that:

1. Resin infiltration effectively masked the demineralization of teeth during orthodontic treatment.
2. Microinvasive WSL infiltration management during orthodontic treatment was found to be superior to topical fluoridation in enhancing the aesthetic appearance of demineralized regions around the brackets.
3. Resin infiltration did not cause clinically visible changes in the adjacent unaffected enamel
4. The esthetic outcome of resin infiltration showed consistent durability at six months.

### Supplementary Information

Below is the link to the electronic supplementary material.

Supplementary file1 (DOCX 42.7 kb)

## Data Availability

The data that support the findings of this study are openly available in figshare at 10.6084/m9.figshare.25584603.v1.

## Declarations

### Trial registration

This trial was registered at the German Clinical Trials Register (DRKS-ID: DRKS00027344).
