# Supplementary material for: Resin infiltration versus fluoride varnish for visual improvement of white spot lesions during multibracket treatment. A randomized-controlled clinical trial
Source: Clin Oral Investig. 2024 May 11;28(6):308. doi: 10.1007/s00784-024-05695-2 (PMC11088558; doi:10.1007/s00784-024-05695-2)
Supplement: Supplementary file 1 — Supplementary file1 (DOCX 42.7 kb) [file 784_2024_5695_MOESM1_ESM.docx]

*CONSORT 2010 checklist of information to include when reporting a randomised trial*

| Section/Topic | Item No | Checklist item | Reported on page No |
| --- | --- | --- | --- |
| *Title and abstract* |  |  |  |
|  | 1a | Identification as a randomized trial in the title | 1 |
|  | 1b | Structured summary of trial design, methods, results, and conclusions (for specific guidance, see CONSORT for abstracts) | 1 |
| *Introduction* |  |  |  |
| Background and objectives | 2a | Scientific background and explanation of the rationale | 1-2 |
|  | 2b | Specific objectives or hypotheses | 2 |
| *Methods* |  |  |  |
| Trial design | 3a | Description of trial design (such as parallel, factorial) including allocation ratio | 2 |
|  | 3b | Important changes to methods after trial commencement (such as eligibility criteria), with reasons | n/a |
| Participants | 4a | Eligibility criteria for participants | 2 |
|  | 4b | Settings and locations where the data were collected | 2-3 |
| Interventions | 5 | The interventions for each group with sufficient details to allow replication, including how and when they were administered | 2-3 |
| Outcomes | 6a | Completely defined pre-specified primary and secondary outcome measures, including how and when they were assessed | 2-3 |
|  | 6b | Any changes to trial outcomes after the trial commenced, with reasons | n/a |
| Sample size | 7a | How sample size was determined | 2 |
|  | 7b | When applicable, explanation of any interim analyses and stopping guidelines | n/a |
| Randomization: |  |  |  |
| Sequence  generation | 8a | Method used to generate the random allocation sequence | 2 |
|  | 8b | Type of randomization; details of any restriction (such as blocking and block size) | 2 |
| Allocation  concealment  mechanism | 9 | Mechanism used to implement the random allocation sequence (such as sequentially numbered containers), describing any steps taken to conceal the sequence until interventions were assigned | 2 |
| Implementation | 10 | Who generated the random allocation sequence, who enrolled participants, and who assigned participants to interventions | 2 |
| Blinding | 11a | If done, who was blinded after assignment to interventions (for example, participants, care providers, those assessing outcomes), and how | 2 |
|  | 11b | If relevant, a description of the similarity of interventions | n/a |
| Statistical methods | 12a | Statistical methods used to compare groups for primary and secondary outcomes | 3 |
|  | 12b | Methods for additional analyses, such as subgroup analyses and adjusted analyses | n/a |
| *Results* |  |  |  |
| Participant flow (a diagram is strongly recommended) | 13a | For each group, the number of participants who were randomly assigned received the intended treatment and were analyzed for the primary outcome | 6 |
|  | 13b | For each group, losses and exclusions after randomization, together with reasons | 6 |
| Recruitment | 14a | Dates defining the periods of recruitment and follow-up | 3 |
|  | 14b | Why the trial ended or was stopped | 3 |
| Baseline data | 15 | A table showing baseline demographic and clinical characteristics for each group | 6 |
| Numbers analyzed | 16 | For each group, the number of participants (denominator) included in each analysis and indicated whether the analysis was by originally assigned groups | 6 |
| Outcomes and estimation | 17a | For each primary and secondary outcome, results for each group and the estimated effect size and its precision (such as a 95% confidence interval) were provided | 3-4,7-8 |
|  | 17b | For binary outcomes, presentation of both absolute and relative effect sizes is recommended | n/a |
| Ancillary analyses | 18 | Results of any other analyses performed, including subgroup analyses and adjusted analyses, distinguishing pre-specified from exploratory | n/a |
| Harms | 19 | All significant harms or unintended effects in each group (for specific guidance, see CONSORT for harms) | n/a |
| *Discussion* |  |  |  |
| Limitations | 20 | Trial limitations, addressing sources of potential bias, imprecision, and, if relevant, multiplicity of analyses | 8 |
| Generalisability | 21 | Generalisability (external validity, applicability) of the trial findings | 5-8 |
| Interpretation | 22 | Interpretation consistent with results, balancing benefits and harms, and considering other relevant evidence | 5-8 |
| *Other information* |  |  |  |
| Registration | 23 | Registration number and name of trial registry | 9 |
| Protocol | 24 | Where the full trial protocol can be accessed, if available | n/a |
| Funding | 25 | Sources of funding and other support (such as supply of drugs) and the role of funders | 9 |

Citation: Schulz KF, Altman DG, Moher D, for the CONSORT Group. CONSORT 2010 Statement: updated guidelines for reporting parallel group randomized trials. BMC Medicine. 2010;8:18.
© 2010 Schulz et al. This is an Open Access article distributed under the terms of the Creative Commons Attribution License (<http://creativecommons.org/licenses/by/2.0>), which permits unrestricted use, distribution, and reproduction in any medium, provided the original work is properly cited.
